# SPINK7 expression changes accompanied by HER2, P53 and RB1 can be relevant in predicting oral squamous cell carcinoma at a molecular level

**DOI:** 10.1038/s41598-021-86208-z

**Published:** 2021-03-25

**Authors:** Gina Pennacchiotti, Fabio Valdés-Gutiérrez, Wilfredo Alejandro González-Arriagada, Héctor Federico Montes, Judith Maria Roxana Parra, Valeria Andrea Guida, Silvina Esther Gómez, Martin Eduardo Guerrero-Gimenez, Juan Manuel Fernandez-Muñoz, Felipe Carlos Martin Zoppino, Rubén Walter Carón, Marcelo Eduardo Ezquer, Ricardo Fernández-Ramires, Flavia Alejandra Bruna

**Affiliations:** 1 Area Medicina Oral Departamento de Patología Facultad de Odontología, Universidad de Chile, Av. Olivos 243, Independencia, Región Metropolitana, CP8320000 Santiago, Chile; 2 Instituto Nacional del Cáncer, Av. Profesor Zañartu 1010, Independencia, Región Metropolitana, CP8320000 Santiago, Chile; 3 CIICOM, Facultad de Odontología, Universidad de Valparaíso, Subida Carvallo 211, Playa ancha, CP2390302 Valparaíso, Chile; 4 Departamento de Cirugía Cabeza-Cuello Clínica Montes, Ituzaingo 1446, CP5500 Capital, Mendoza, Argentina; 5 Servicio de Medicina Bucal (SEMEB), Facultad de Odontología, Universidad Nacional de Cuyo (FOdonto-UNCuyo), Centro Universitario, CP5500 Mendoza Capital, Argentina; 6 Instituto de Medicina y Biología Experimental de Cuyo (IMBECU) CCT-Mendoza CONICET, Universidad Nacional de Cuyo (UNCuyo). Av. Adrián Ruiz Leal W/N, CP5500 Capital, Mendoza, Argentina; 7 Centro de Medicina Regenerativa, Facultad de Medicina, Universidad del Desarrollo-Clínica Alemana (FM, UDD-CAS), Av Las Condes 12.438, CP7690000 Lo Barnechea. Santiago, Chile; 8 Escuela de Odontología, Facultad de Ciencias, Universidad Mayor (UMayor), Avda Libertador Bernardo O`Higgins 2013. Región Metropolitana, CP8320000 Santiago, Chile

**Keywords:** Cancer, Cell biology, Molecular biology, Biomarkers, Molecular medicine, Oncology

## Abstract

The oral squamous cell carcinoma (OSCC), which has a high morbidity rate, affects patients worldwide. Changes in SPINK7 in precancerous lesions could promote oncogenesis. Our aim was to evaluate SPINK7 as a potential molecular biomarker which predicts OSCC stages, compared to: HER2, TP53, RB1, NFKB and CYP4B1. This study used oral biopsies from three patient groups: dysplasia (n = 33), less invasive (n = 28) and highly invasive OSCC (n = 18). The control group consisted of clinically suspicious cases later to be confirmed as normal mucosa (n = 20). Gene levels of *SPINK7, P53, RB, NFKB* and *CYP4B1* were quantified by qPCR. *SPINK7* levels were correlated with a cohort of 330 patients from the TCGA. Also, SPINK7, HER2, TP53, and RB1, were evaluated by immunohistofluorescence. One-way Kruskal–Wallis test and Dunn's *post-hoc* with a *p* < 0.05 significance was used to analyze data. In OSCC, the *SPINK7* expression had down regulated while *P53, RB, NFKB* and *CYP4B1* had up regulated (*p* < 0.001). *SPINK7* had also diminished in TCGA patients (*p* = 2.10e-6). In less invasive OSCC, SPINK7 and HER2 proteins had decreased while TP53 and RB1 had increased with respect to the other groups (*p* < 0.05). The changes of SPINK7 accompanied by HER2, P53 and RB1 can be used to classify the molecular stage of OSCC lesions allowing a diagnosis at molecular and histopathological levels.

## Introduction

Oral squamous cell carcinoma (OSCC) is the most common malignancy of the head and neck. The morbidity rate of OSCC remains high even after five years of been diagnosed (37.8%)^1^. Despite great improvement in treatment and therapy, the prognosis remains poor^2^. Furthermore, OSCC often causes dysfunctions and aesthetic disorders. It also has a high incidence in cervical lymph node metastasis thus worsening patients’ quality of life^3^. Several tumor biomarkers have been suggested as predictive for OSCC prognosis with poor outcome^4^, however, specific molecular prognostic factors have only been partially identified^5^. The pattern of invasion (POI) presented by Brandwein-Gensler et al., classifies the POI in five types^6^, and has been validated as an independent prognostic factor in oral cancer^7^. However, it is necessary to identify changes in proteins and genes, to improve diagnostic strategies in precancerous, invasive and metastatic stages^8^.

One of the cancer hallmarks is the alteration of molecules related to cell adhesion and migration^9^. Adhesion molecules play a central role in pathogenesis and progression of malignant tumors^10^. Serine Peptidase Inhibitor, Kazal Type 7 (SPINK7, ECRG2) belongs to a family of 13 members (1–13) of proteins with inhibitory Serine Peptidase activity identified in 1998 in esophageal tissue^11^. This *novel* tumor suppressor gene was identified as such by comparing normal esophageal epithelia and primary squamous cell carcinoma tissues^12,13^. It has been reported that SPINK7 inhibits tumor cell growth, promotes cell apoptosis, and inhibits cancer cell migration, invasion and metastasis in vitro^14,15^.

The HER2, P53, RB1, NFKB and CYP4B1 genes and their proteins were found to be altered in OSCC carcinogenesis^16^. The human epidermal growth factor receptors (HER/EGFR) are a family of trans membrane tyrosine kinase receptors comprising 1 to 4 (HER1-4)^17^. The overexpression of HER is involved in the development of oncogenesis, including OSCC, as it regulates different cellular pathways. HER2 (also known as C-erbB-2/ERBB2/ErbB2) plays a critical role in cell proliferation, survival, migration, angiogenesis, and metastasis through a variety of intracellular signaling cascades such as MAPK/ERK1/2 and Pi3K/Akt^18,19^. An imbalance in these pathways can lead to permanent activation^20,21^. Studies have established a marked correlation between HER2 expression and the poor survival of OSCC patients^22^. It has been reported that the SPINK proteins family share 50% of homology to the EGF protein and can interact by binding with the EGFR receptor thus activating EGFR downstream AKT signaling pathway, inducing epithelial mesenchymal transition^23^. The SPINK6 protein is secreted and acts as a functional regulator of nasopharyngeal carcinoma cell metastasis through the binding to the EGFR extracellular domain^23^.

In cancer cells, tumor-suppressor genes like Protein 53 (TP53) and Retinoblastoma (RB1) are inactivated by mutation, deletion and methylation^24^. It is well established that TP53 is a genome guardian and plays a pivotal role in regulating the cell cycle, cellular differentiation, DNA repair, and apoptosis^25,26^. Somatic mutations in TP53 are detected in > 60% of OSCC and in 10% of oral dysplasia^27^. Recently, Genome Wide Association Study data has shown that TP53 is usually mutated in papillomavirus-negative OSCC patients^28^. The TP53 mutations in OSCC (classified in low- and high-risk missense mutations) are associated with resistance to Cisplatin, distant metastasis and poor prognosis^29–31^. The overall survival of TP53-mutant OSCC patients is also markedly worse than patients with TP53 wild-type^32^. Previous studies reported that SPINK7 also participates in centrosome amplification in a TP53-dependent manner and has a role in maintaining chromosome stability^33^. RB1, another gene that plays a key role in the regulation of cell cycle and differentiation was found to be altered in OSCC. The active form of RB1 (phosphorylated pRB1), acts a regulator at the G1-S restriction point as it arrests the cell cycle^34^. Mutations lead to functional pRB1 inactivation, failure of growth and tumor suppression control^35^.

Another molecule altered in carcinogenesis is the Nuclear factor-κB (NFKB). This is a proinflammatory transcription factor that plays a pivotal role in the initiation and progression of the cancer^36^. NFKB is constitutively activated in OSCCs and is involved in promoting the invasive characteristics^37^. The cytochrome P450 enzyme family (CYP450) is one of the most important with respect to the cell detoxification machinery^38,39^. Their activity consists in catalyzing reactions that participate in both biosynthesis and degradation of drug metabolism and xenobiotic biotransformation pathways^40^. These enzymes can participate indirectly in the OSCC carcinogenesis through activation and detoxification of these compounds^38,40^.

The Cancer Genome Atlas (TCGA) is an important tool to provide expression profiles from cancer patient samples and the associated clinical-pathological data for > 30 human cancer types^41^. However, there are few studies on genome-wide profiling of OSCC tumors.

The aim of this work was to evaluate SPINK7 as a potential molecular biomarker which predicts OSCC stages, compared to: HER2, TP53, RB1, NFKB and CYP4B1, as well to explore their potential therapeutic applications (early detection and targeted therapies).

## Results

### Study population data

Of a total of 71 Caucasian patients with oral dysplasia or OSCC consented and were enrolled in the present study. The average age was 52 years. The predominant gender was male. Of the total of patients, 30% had no smoking habits, 20% were light smokers (less than 10 cigarettes per day), 40% heavy smokers (more than 10 cigarettes per day) and a 10% did not specify if they had smoking habits; the tongue ride being the most frequent tumor location (80%) (Table 1). The supplementary Table 2 shows the TNM, POI and deep invasion of the epithelium score respect to the histopathological diagnosis.

**Table 1:** Sociodemographic table of the study population.

Population of study data	Number of patients with OSCC lesions	Percentage of patients with OSCC lesions (%)
**Age**		
< 40 years old	12	15
41–59 years old	32	40
> 60 years old	37	45
**Gender**		
Female	32	40
Male	49	60
**Smoke habits**		
No	24	30
Light smokers (< 5/dia)	16	20
Heavy smokers (> 10/dfa)	41	40
Non specific	1	10

### The oral epithelium changes among OSCC stages

**Figure 1:**
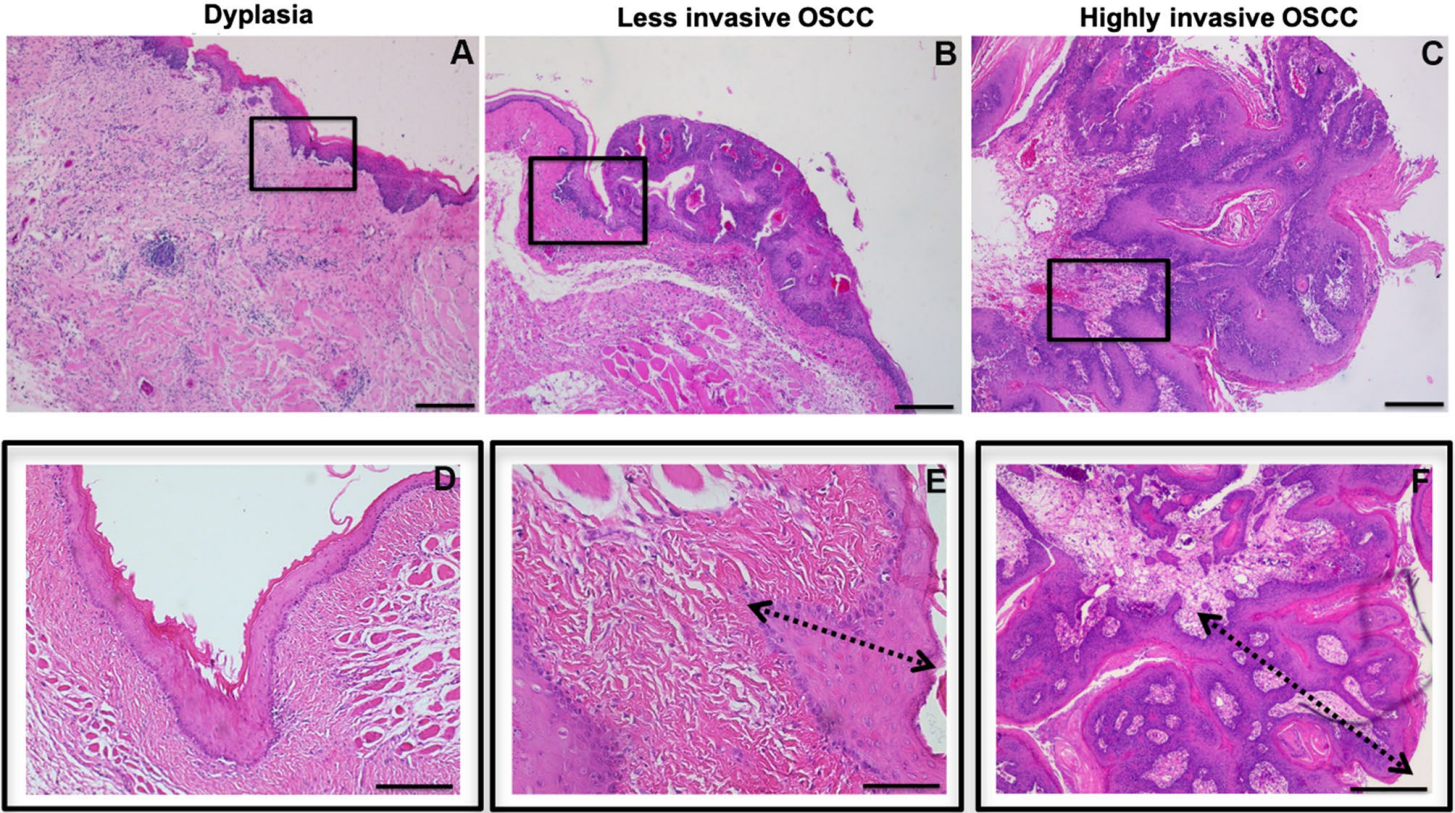
OSCC stages H&E analysis. Oral biopsies of patients were analyzed by H&E and classified into dysplasia (**A** and **D**), less invasive OSCC (**B** and **E**) and highly invasive OSCC (**C** and **F**) according to the changes in the epithelium. The (**A**) and (**D**) show the dysplasia histology at different magnifications. The tissue did not show loss of basal membrane continuity or presence of epithelial cells invading the stroma, although an increase in epithelial cell layers and presence of mild leukocyte infiltrate was observed. The biopsies of patients with OSCC both less (**B** and **E**) and highly invasive cases (**C** and **F**), at different magnification showed more than 5 layers of epithelial cells, hyperchromatism, presence of keratin pearls, loss of continuity of the basement membrane and severe leukocyte infiltrate were seen. In the highly invasive OSCC group, epithelial cell nests were observed in the stroma and severe epithelium disorganization, accompanied of leucocyte infiltration of high grade (**C**-**F**).

To analyze epithelial changes in OSCC progression, the oral biopsies were evaluated by H&E and classified in dysplasia, less invasive and highly invasive OSCC. We found 33 cases of dysplasia, 28 cases of less invasive OSCC and 18 cases of highly invasive OSCC. In all dysplasia cases, the tissue did not show loss of basement membrane continuity or presence of epithelial cells invading the stroma, although an increase in epithelial cell layers and presence of mild leukocyte infiltrate was observed (Fig. 1A–D). In the OSCC groups both (less invasive and highly invasive), showed more than 5 layers of epithelial cells, hyperchromatism, cellular atypia and presence of keratin pearls, loss of continuity of the basement membrane and a severe leukocyte infiltrate was observed (Fig. 1B, C, E, F). In the highly invasive OSCC group, epithelial cell nests were observed in the stroma, total epithelial disorganization, keratin pearls and the leucocyte infiltration was severe. These results were correlated with poor prognosis. The inserts (black square) at high magnification show the oral epithelium changes among the OSCC stages (Fig. 1D–F). The oral epithelial cells alterations among less and highly invasive OSCC was showed in the supplementary Fig. 1.

### SPINK7 generates a distinctive molecular signature among the OSCC stages

**Figure 2:**
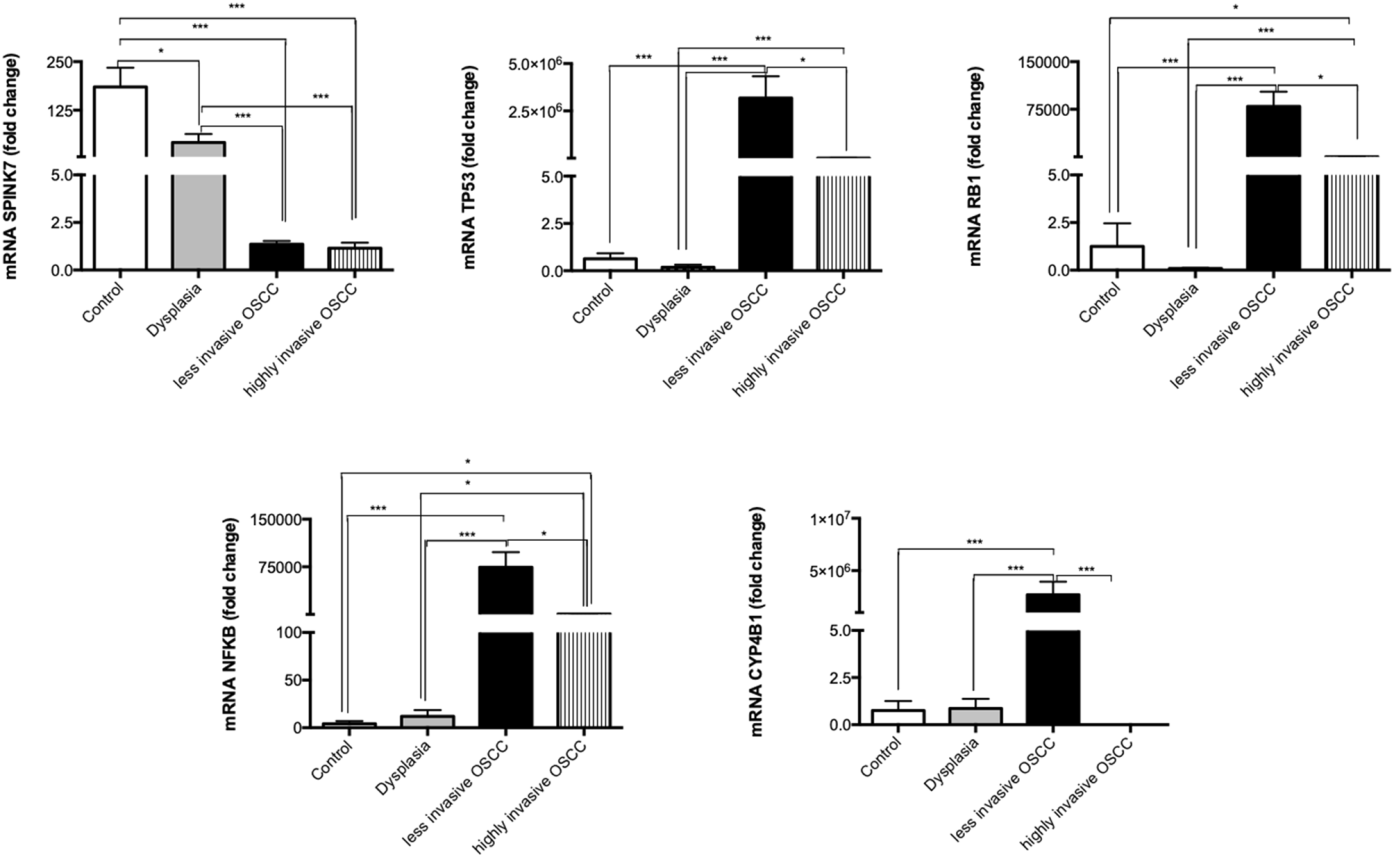
*SPINK7, TP53, RB1, NFKB* and *CYP4B1* gene expression changed among the OSCC stages. The bar graph showed the gene expression evaluated by qPCR of each group (normal, dysplasia, less invasive and highly invasive OSCC) and the results were expressed as arbitrary units. The differences were considered statistically significant with *P* values of (*p < 0.05, **p < 0.01 and ***p < 0.001). Comparisons test was performed using GraphPad Prism version 6.00c for Windows, GraphPad Software, La Jolla California USA, www.graphpad.com”.

To evaluate the molecular status of the biopsies among the OSCC stages, we assessed the gene expression of *SPINK7* with reported altered genes in carcinogenesis: *TP53, RB1, NFKB* and *CYP4B1* in the groups. We found that *SPINK7* progressively down regulated in oral dysplasia and OSCC groups with respect to the control group (*p* < 0.001). Regarding *TP53, RB1, NFKB* and *CYP4B1* all were found to be up regulated in OSCC groups with respect to dysplasia and control groups (*p* < 0.001). Additionally, with the exception of *SPINK7*, we observed differential expression levels of the rest of the genes between less invasive OSCC and highly invasive OSCC groups (*TP53, RB1* and *NFKB* (*p* < 0.05); *CYP4B1* *p* < 0.001)) (Fig. 2). The differences observed regarding the gene expression among the groups were correlated with the prognosis (less versus highly invasive OSCC).

### SPINK7 was found to be down regulated in patients from TCGA

**Figure 3:**
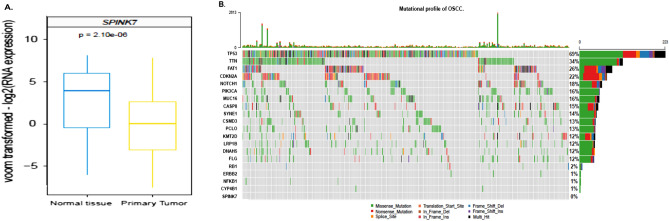
*SPINK7* gene expression and mutational profile in a cohort of patients from the TCGA database. (**A**) Box-plot derived of TCGA gene expression analysis of *SPINK7* from 581 samples of the normal group (blue box-plot) vs the primary tumors group (yellow box-plot). (**B**) The Oncoplot graph shows the profile of oral cancer mutations taking into account the fifteen genes with the highest number of mutations (SNP) followed by five genes of interest (*RB1, ERBB2/HER2, NFKB1, CYP4B1* and *SPINK7*). Each column represents a sample of oral cancer and each color represents a type of mutation variant. On the right, the size of each bar represents the frequency of mutations throughout all samples and the percentage of samples that have this mutated gene. Variants annotated as Multi_Hit are those genes that mutated more than once in the same sample. In the upper part shows the number of mutations per sample.

To evaluate if the *SPINK7* gene expression profile observed in our study population was reproducible with other cohorts of OSCC patients, we analyzed the gene expression levels in 581 patients with OSCC from the TCGA (primary tissue) and the results were compared with normal subjects data (normal tissue) through in silico analysis^42^. The results were graphed as a box plot comparing the gene expression of the normal group (blue box plot) versus the primary tumors group (yellow box plot) showing significant down regulation of *SPINK7* *p* = 2.10^e-06^ and this result was correlated with our results respect to *SPINK7* gene expression (Fig. 3A).

### SPINK7 does not show mutations according to the TCGA mutation gene profile

To understand if the differential expression of *SPINK7* among the OSCC stages is related to a mutational profile, we analyzed in silico mutations described to date of a cohort of 329 patients from the TCGA database. We identified 15 genes with a differential rate of mutations associated to OSCC, SPINK7 being the gene of interest included in the analysis. We found that *TP53* was the gene with the highest number of mutations in the OSCC cohort, being mutated in 69% of patients (Fig. 3B). The most frequent type of variant was missense mutations followed by nonsense mutations, frameshift deletions, and multi hits mutations. *TTN* gene showed a mutation rate of 34%, *FAT1* (26%), *CDKN2A* (22%), *NOTCH1* (18%), *PIK3CA* and *MUC16* (16%), *CASP8* (15%), *SYNE1* (14%), *CSMD3* and *PCL2* (13%) and finally, *KMT2D, LRP1B, DNAH5* and *FLG* genes were mutated in 12% of the patients. On the other hand, and with a more stable mutational profile, we found that *RB1* was mutated in 2% of the samples, *HER2, NFKB1* and *CYP4B1* (1%) of the cohort. Finally, we found that *SPINK7* is a genomic stable gene, which showed no mutations in any of the cases analyzed (Fig. 3B).

### SPINK7 and HER2 changed differentially among the OSCC stages

**Figure 4:**
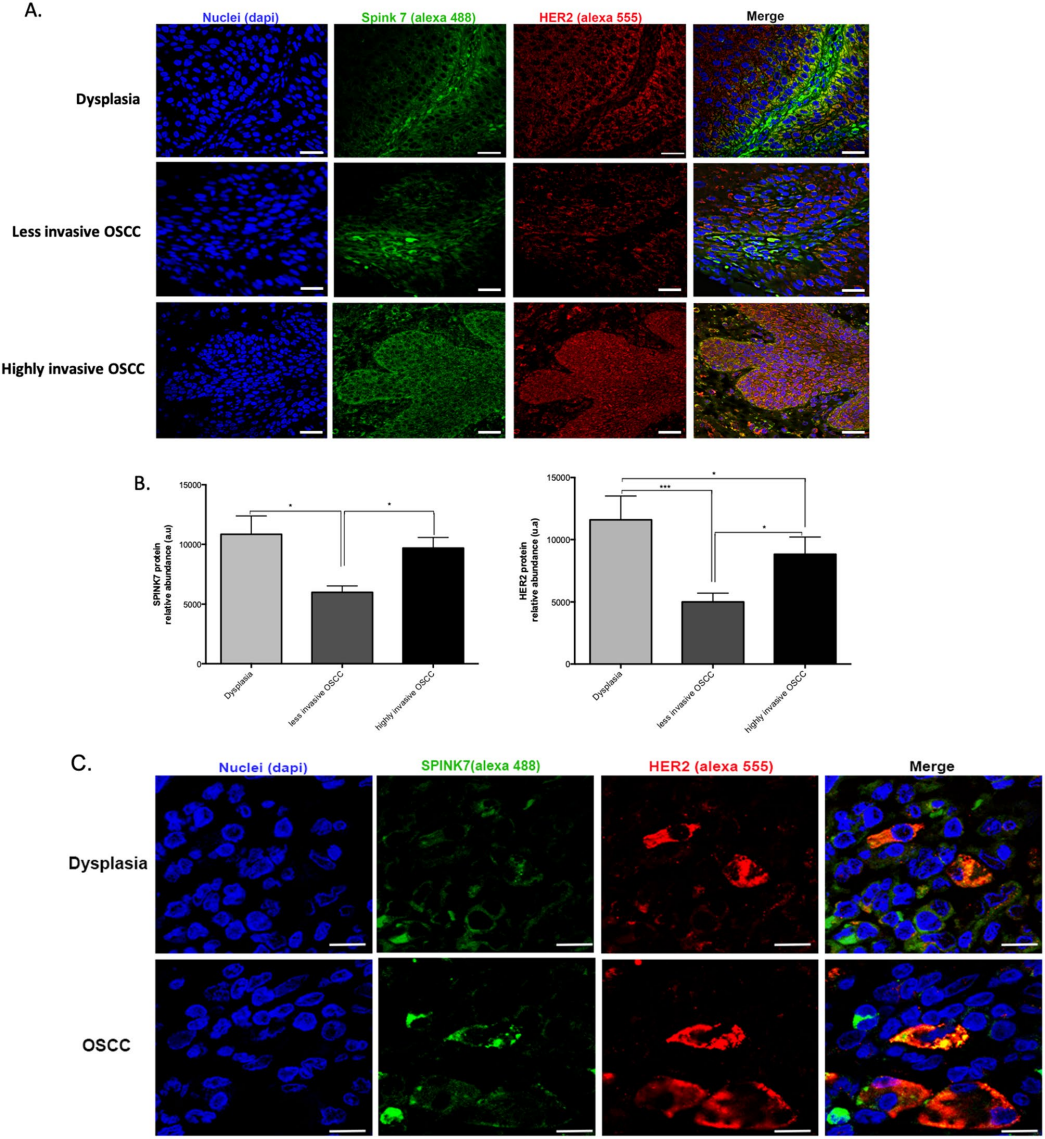
SPINK7 and HER2 proteins analysis among the OSCC stages. (**A**) Immunohistofluorescence of SPINK7 (green) and HER2 (red) proteins evaluated in biopsies of dysplasia, less invasive and highly invasive OSCC. Nuclei were stained with Dapi (blue). The (0**B**) show the bar graphs of the quantitative analysis of pixels intensity (green = SPINK7 and red = HER2) assessed by Image J (version: 2.1.0/1.53c; open The (**C**) show the images at high magnification (120X) among the OSCC stages, the close localization of both proteins signal through overlap images (yellow pixels), Representative images by group, n = 6. White bar = 50 μm. The differences were considered statistically significant with P values of (*p < 0.05 and ***p < 0.001). Comparisons test was performed using GraphPad Prism version 6.00c for Windows, GraphPad Software, La Jolla California USA, www.graphpad.com”. source image processing software Copyright 2010–2021, http://imageJ.net/Contributors).

It has been reported previously that SPINK proteins can interact with a HER2 receptor^43^. We evaluated the presence and abundance of SPINK7 among the different groups and its correlation with HER2 protein by confocal microscopy. We found that SPINK7(green signal) and HER2 (red signal) had significantly decreased in the less invasive OSCC group compared with dysplasia and highly invasive OSCC groups (*p* < 0.05). On the other hand, the highly invasive OSCC group showed SPINK7 protein levels similar to dysplasia and no significant differences were found. Regarding HER2, it was found to be significantly reduced in the less invasive OSCC compared to the other groups. Meanwhile, the highly invasive OSCC group showed a significant increase of HER2 compared to less invasive OSCC (*p* < 0.001), being similar to the dysplasia group (Fig. 4A, B).

### SPINK7 and HER2 were colocalized

Due to the overlapping of signals (yellow signal) between SPINK7 (green signal) and HER2 (red signal) in confocal microscopy, we evaluated at high magnification images (120X digital zoom) through colocalization analysis. The intensity variability of both channels was statistically evaluated using Pearson's coefficient of 1 as positive result (r = 0,99). Yellow versus red and green pixels were quantified yielding a co-occurrence value of 56.58% (Fig. 4C).

### TP53 and pRB1 changed differentially among the OSCC stages

**Figure 5:**
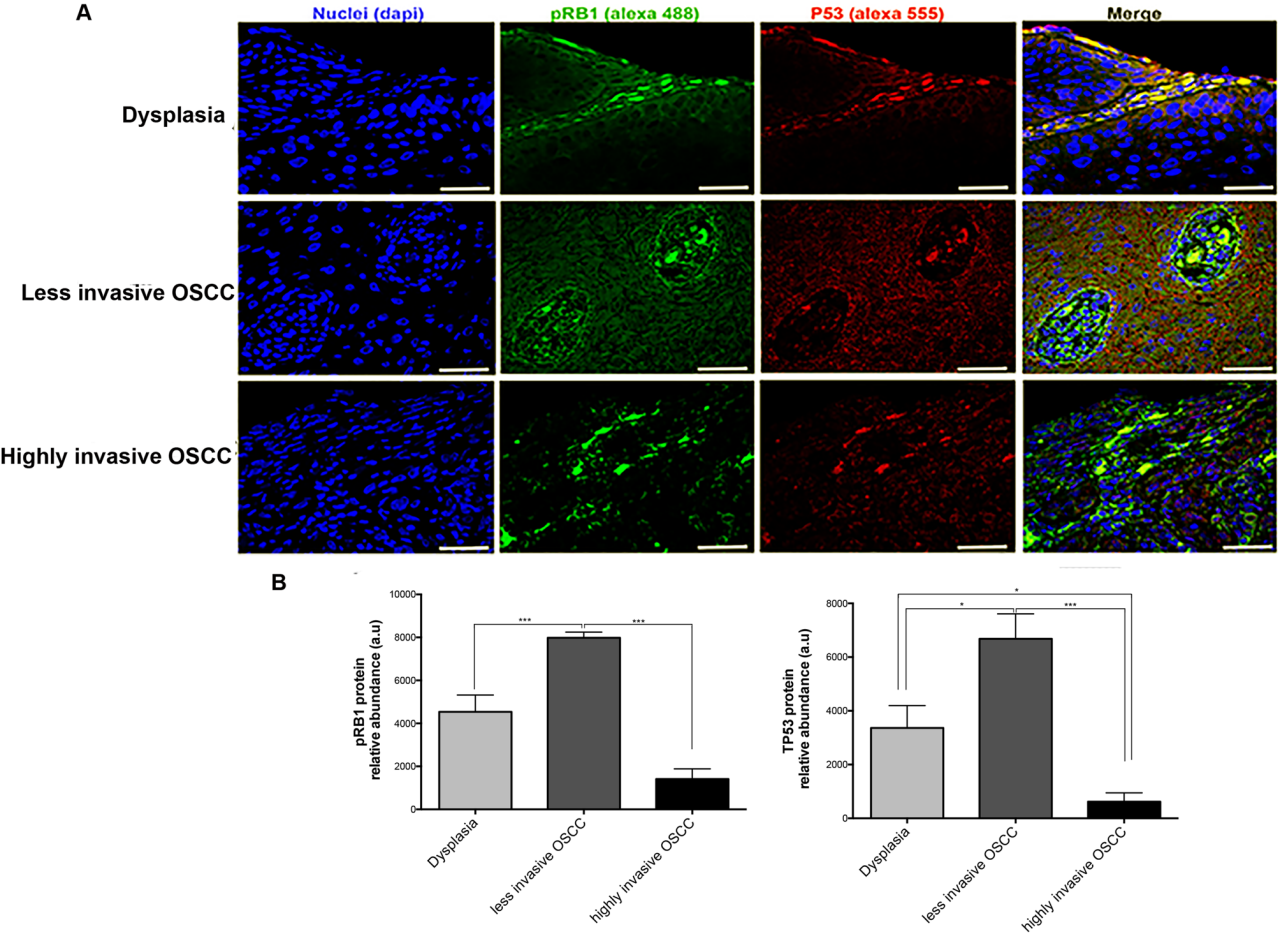
pRB1 and P53 proteins analysis among the OSCC stages. (**A**) Immunohistofluorescence of both proteins, evaluated in biopsies of dysplasia, less invasive and highly invasive OSCC. (**B**) The bar graphs show quantitative analysis of pixels intensity (green = pRB1 and red = P53) assessed by ImageJ (version: 2.1.0/1.53c; open source image processing software Copyright 2010–2021, http://imageJ.net/Contributors). Representative images by group, n = 6/group. White bar = 50 μm. The differences were considered statistically significant with *p* values of (*p < 0.05 and ***p < 0.001). Comparisons test was performed using GraphPad Prism version 6.00c for Windows, GraphPad Software, La Jolla California USA, www.graphpad.com”.

To evaluate cell cycle regulators in the OSCC, we analyzed TP53 and pRB1 protein levels among the groups by confocal microscopy. The less invasive OSCC group showed a significant increase of TP53 and pRB1 compared to dysplasia and highly invasive OSCC groups (*p* < 0.05). Regarding the highly invasive OSCC group, both proteins (TP53 and pRB1) significantly decreased with respect to the other groups (*p* < 0.001) (Fig. 5A, B).

## Discussion

The oral squamous cell carcinoma has a high morbidity rate in the world^1^. Despite the progress in research and therapy, survival has not improved significantly in the last decades^44^. The biomarkers study aims to understand the role of genetic and lifestyle factors of the tumor biology including OSCC^45^. We studied changes in proteins related with some of the cancer hallmarks (cell survival, cell cycle, inflammation, metastasis and metabolism) to stratify molecularly oral precancerous and cancerous lesions^46^. Currently, the gold standard of OSCC diagnosis is the biopsy, however, the results are observer-dependent and subjective^44,47^.

The current study is reporting the SPINK7 expression changes among the OSCC stages and we propose this protein as a "*new biomarker”* associated with the natural progression of the OSCC. We found differences among the oral epithelial organization in dysplasia and less or highly invasive OSCC groups, and these results were correlated with the literature^48,49^, showing a differential gene expression profile by qPCR analysis with a distinctive "molecular signature” in each stage. We found *SPINK7* to be significantly down regulated at dysplasia and OSCC compared with the control group. Meanwhile *TP53, RB1, NFKB and CYP4B1* were found to be significantly up regulated at OSCC stages compared with dysplasia and the control groups. The results obtained by SPINK7 in our study population were compared to a cohort of 541 patients from the TGCA database^50^. The comparative analysis showed that *SPINK7* was found to be significantly down regulated in patients with OSCC compared to normal tissue and this could be related with the advanced stage of the malignant lesion^51^. Additionally, we analyzed the mutation profile of genes described in TCGA altered in OSCC including *SPINK7, TP53, RB1, NFKB* and *CYP4B1*. We found that *TP53* showed a high mutation rate in OSCC meanwhile *SPINK7* was the most stable without any mutation described. These results suggest that the down regulation of the gene would be related to other mechanisms not associated to *TP53* gene and needs to be explored in the future.

Due to the fact that the SPINK proteins family is related with extracellular matrix remodeling and cell migration regulation^43^, we evaluated the abundance of SPINK7 and HER2 and if there is correlation between them. It has been reported that SPINK7 shares 50% of homology with EGF^43^. We found SPINK7 increased in the highly invasive OSCC group. These results were similar to previous studies describing that SPINK6 was increased in highly metastatic tumors^43^. SPINK6 regulates the metastasis via EGFR signaling and their expression levels change during the carcinogenesis^52^. Interestingly, SPINK7 and HER2 were overexpressed in the highly invasive OSCC compared to the less invasive OSCC group. Additionally, the SPINK7 and HER2 colocalization analysis showed that both proteins are close (suggesting interaction), however subsequent tests with a larger sample size are necessary to evaluate and understand its interaction or co-compartmentalization^51^. The differential expression of the studied proteins among the OSCC stages could be related with disorganization of the oral epithelium and to a non-functional protein or absence of their ligands, but it needs to be explored in more detail in the future^53^. The differential proteins expression among the stages allowed stratifying the groups to molecular and histological levels correlated with prognosis. It has been reported in esophageal cancer that cells treated with a siRNA for SPINK1 were resistant to the antitumoral drug Cisplatin^54^. This could be interesting in order to stratify the patients who respond, or not, to the standard chemotherapy.

Regarding the studied cell cycle factors, TP53 and pRB1, the confocal microscopy analysis showed that both proteins up regulated in the less invasive OSCC respect to dysplasia; meanwhile in the highly invasive OSCC both down regulated, which is consistent with previous studies^55^. These could be explained through the high rate of mutation profile of both genes showed in OSCC in the TCGA in silico analysis. These results suggest that in OSCC, TP53 and pRB1 are present but non-functional and this could be related with a more aggressive tumor^55^. We found that SPINK7 had significantly downregulated in the less invasive OSCC group, while TP53 levels remained higher. This could be explained in part by the results recently published by Patel, H. et al., which showed that SPINK7 is a novel transcriptional target of p53 and can modulate cancer cell sensitivity to DNA damage in cell death and disease. Interestingly, while wild-type p53 increase SPINK7 protein levels, tumor-derived mutant p53 (R273H) does not change or decrease SPINK7 protein levels.^56^.

Our results suggest that the changes in the expression of SPINK7 can be used to predict the molecular stage of the OSCC lesions. This molecule could be a new “potential” biomarker. Future studies are needed to validate this *novel* tumor suppressor gene that could be applied as a possible early diagnostic method to precancerous oral lesions and OSCC.

## Methods

### Study population

Patients with suspected oral lesions of OSCC were enrolled. After signing the informed consent, the subjects were interviewed using a standard questionnaire that requested information about socio-demographic, medical, and lifestyle factors. The patients from Department of Head and Neck surgery of The National Cancer Institute, Dental school of Universidad de Valparaíso, Dental school of Universidad del Desarrollo (Chile), The Hospital Lencinas and the Servicio de Estomatología y Medicina Bucal Dental school, Universidad Nacional de Cuyo (Argentina); received a routine intraoral examination and oral mucosal biopsies were taken and classified according to the diagnosis and POI in three groups: oral epithelial dysplasia, less invasive OSCC (POI type 1 and 2) and invasive OSCC (POI type 3, 4 and 5) group. Eighty-one cases of primary OSCC diagnosed over a period of 2 years (2017–2019) were included in the study. None of the patients had received any tumor specific therapy (chemotherapy or radiotherapy) before the resection. Twenty cases diagnosed as inflammatory lesions and histologically confirmed with normal mucosal margins from the resection specimens were included as control group in the qPCR analysis. The Ethics Committee of the School of Medicine of Universidad del Desarrollo (FM-UDD CAS), National Cancer Institute of Chile and Medicine School of Universidad Nacional de Cuyo (FCM-UNCuyo) approved this study according to Declaration of Helsinki to experimentation with human subjects.

### Histopathological analysis

The Oral biopsies were fixed in 10% buffered formalin (Merck, USA), embedded in paraffin (Merck), and sectioned. Tissue sections of 4 μm were deparaffinized with Neoclear (Merck), rehydrated with graded alcohols, stained with hematoxylin–eosin (H&E, Merck), and visualized with a light microscope (DM2000; Leica, Germany). Images were captured with a digital camera (DFC295; Leica). Samples were classified according to the revised criteria given by the World Health Organization (2005). Three independent observers performed histological analyses blind; one of them is a pathologist expert in oral diseases^57,58^.

### Immunohistofluorescence analysis

Tissue sections of 4 μm were deparaffinized, rehydrated, blocked with 5% FBS (Gibco, USA) dissolved in PBS 1X (Gibco, USA) and incubated overnight at 4 °C with a dilution 1:50 of antibodies for anti-SPINK7 (Abcam, ab122326, USA), anti-HER2 (BD Pharmigen™, #554,299, USA), anti-p53 (Abcam, (PAb 1801 ab28, USA) and anti-pRB (8516S, Cell signaling, USA). Then, samples were washed with PBS 1X and incubated two hours at room temperature with a dilution 1:400 of Alexa488-conjugated goat anti-mouse IgG or Alexa 555-conjugated rabitt anti-mouse IgG (Cell Signaling, USA). Cross-reactivity of the secondary antibody was tested incubating samples without the primary antibody. Nuclei were counterstained with a dilution 1:1500 of DAPI (Sigma, Aldrich) in PBS 1X. Samples were embedded in fluorescence mounting medium S3023 (Dako cytomation, USA) and scanned in a confocal microscope (Olympus). Five representative optical sections by sample (n = 6/group) were photographed using 60X magnification. The images obtained per field of each sample, were processed with the same conditions and the positive protein signal (pixels intensity) was analyzed and quantified using Fiji Image J software (NIH, USA)^57,58^.

### Confocal microscopy analysis

A Gaussian filter of 1 was applied and a constant background value of 150 was subtracted for each image. The same threshold value was set for each channel including the structures of interest and the corresponding masks were obtained. The yellow pixels (red and green pixels overlap) versus the total pixels were quantified and the colocalization was measured with Coloc2 plugin (Fiji ImageJ)^51^.

### Gene expression analysis

Total RNA was isolated from the oral biopsies. The mRNA was purified using RNEasy PlusMini Kit (Qiagen, Germany). Contaminating genomic DNA was degraded with 1 U of DNAse RQ1 (Promega). One μg of RNA was reverse transcribed for 60 min at 42 °C using 200 U M-MLV reverse transcriptase (Invitrogen) and 0.5 μM oligo-dT primers (Invitrogen). Real time PCR was performed in a final volume of 10 μL containing 50 ng of cDNA, Power SYBR Green PCR master mix (Life Technologies, Grand Island, NY) and 0.5 μM of each specific primer, using the Step One Plus PCR system (Life Technologies). Controls without reverse transcriptase were included. Amplicons were analyzed according to their size and melting temperature (Supplementary Table 1). To normalize data, 18S RNA and β-actin were used as reference genes. The RNA level of a target gene was calculated using the 2ΔCt method and graphed as fold change^59^.

### Gene expressions TCGA profile

The data studied was programmatically extracted from the publicly available data set of OSCC from The Cancer Genome Atlas Project (TCGA) on May, 2019 using the recount2 platform (https://jhubiostatistics.shinyapps.io/recount/). Non-standardized RNASeq gene expression levels from 548 samples were downloaded. Samples from oral cavity were selected obtaining a final subset of 332 tumor samples and 32 non-tumoral tissue samples. RNA expression levels were evaluated for 6 genes (NFKB1, RB1, TP53, ERBB2, CYP4B1, SPINK7). Crude counts were scaled by the total coverage of the sample (area under the curve, ‘AUC’) and differential gene expression analysis (DGE) was performed using the generalized linear model method of the EdgeR R package comparing non-tumor versus tumor samples^60^. Log_2_ Fold change values were obtained associated with exact p-values and False Discovery Rate values (FDR). To evaluate gene expression correlation, data was transformed using Voom conversion from the R limma package, allowing normal linear modeling of the RNA counts. Afterwards, pairwise Pearson’s product-moment correlation analysis was performed for the aforementioned genes and p-values were calculated^50^.

### Gene mutations TCGA profile

The mutational analysis of OSCC, data was programmatically downloaded using the TCGA biolinks package of Bioconductor^61^. Mutation Annotation Format (MAF) files with aggregated mutation information generated from whole-exome sequencing were downloaded. From 546 samples of Head and Neck cancer, 329 samples of OSCC were obtained. The maftools Bioconductor package was used to analyze and visualize the MAF files^62^. An Oncoplot was drawn showing the variants (SNP) of the 15 most mutated genes in OSCC, followed by 5 genes of interest (RB1, ERBB2, NFKB1, CYP4B1 and SPINK7)^50^.

### Statistical analysis

The population distribution of the samples from our patients was non-parametric. Comparisons of gene and protein expression among the groups were performed using One-way Kruskal–Wallis test and Dunn's test as post-test. Stat Graph Prism 6.0c software was used for statistical analysis. Data are presented as median ± SEM, and *p* < 0.05 was considered statistically significative.

### Ethics approval and consent to participate

The Ethics Committee of the School of Medicine of Universidad del Desarrollo (FM-UDD CAS), National Cancer Institute of Chile and Medicine School of Universidad Nacional de Cuyo (FCM-UNCuyo) approved this study according to Declaration of Helsinki to experimentation with human subjects.

## Supplementary Information

Supplementary Information
