# Supplementary material for: SPINK7 expression changes accompanied by HER2, P53 and RB1 can be relevant in predicting oral squamous cell carcinoma at a molecular level
Source: Sci Rep. 2021 Mar 25;11:6939. doi: 10.1038/s41598-021-86208-z (PMC7994578; doi:10.1038/s41598-021-86208-z)
Supplement: Supplementary file 1 — Supplementary Information [file 41598_2021_86208_MOESM1_ESM.docx]

**SPINK7 expression changes accompanied by HER2, P53 and RB1 can be relevant in predicting oral squamous cell carcinoma at a molecular level**

Gina Pennacchiotti, DMD; Fabio Valdés Garrido, MD; Wilfredo A González-Arriagada, PhD; Héctor F Montes, MD; Judith MR Parra, DMD; Valeria A Guida, DMD; Silvina E Gómez, Bsc; Martin E Guerrero-Gimenez, MD; Juan M Fernandez-Muñoz, Bsc^6^; Felipe CM Zoppino, PhD^6^; Rubén W Carón, PhD; Marcelo E Ezquer, PhD; Ricardo Fernández-Ramires, PhD and Flavia A Bruna, PhD.

**Pennacchiotti et al, Supplementary Table 1**

**
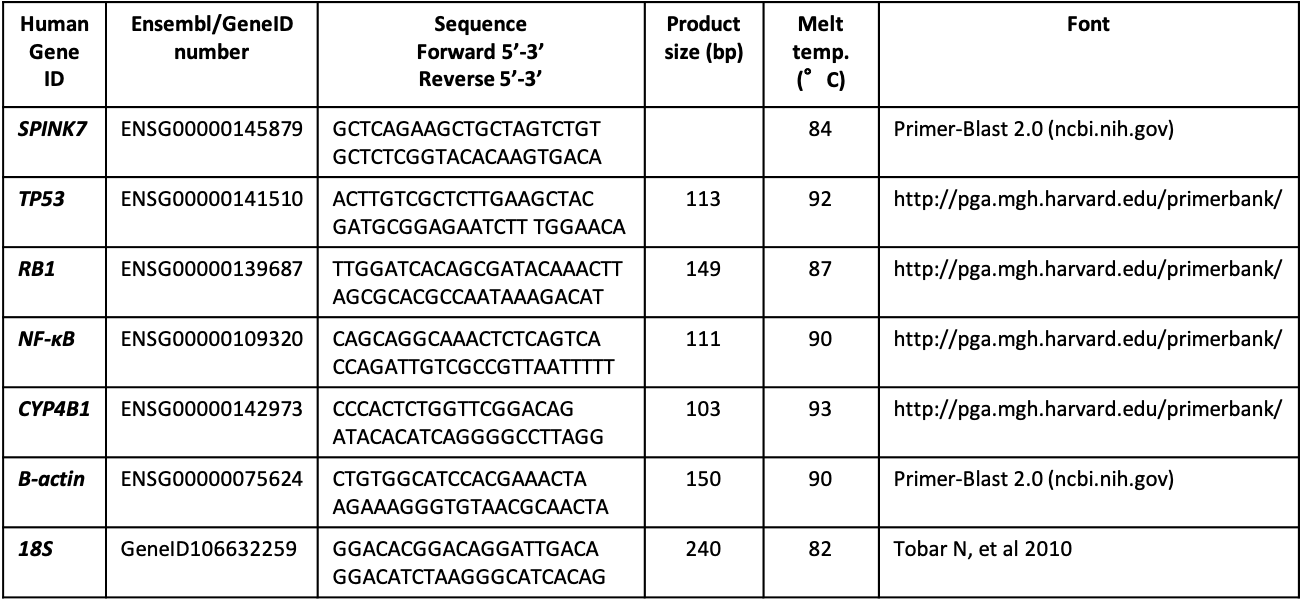
**

**Pennacchiotti et al, Supplementary Table 2**

**
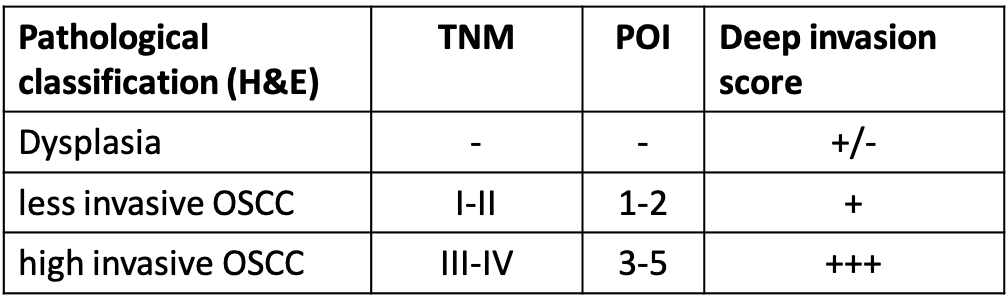
**

**Pennacchiotti et al, Supplementary figure 1 and legend**

**
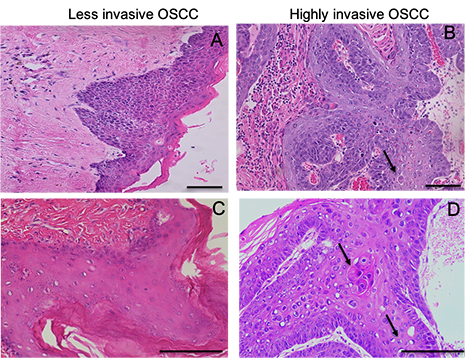
**

**Supplementary Figure 1. H&E analysis of less invasive versus highly invasive OSCC.** Oral biopsies of patients were analyzed by H&E and classified into less invasive or highly invasive OSCC according to the architecture changes. The representative images show hyperchromatism, cellular atypia (arrows), loss of continuity of the basement membrane, deep invasion of the epithelium into the stroma. The OSCC invasive group showed a total epithelium disorganization this was correlated with poor prognosis (Figure S1A-D).
